# Role of Hypertension in Aggravating A*β* Neuropathology of AD Type and Tau-Mediated Motor Impairment

**DOI:** 10.1155/2009/107286

**Published:** 2009-09-17

**Authors:** C. Díaz-Ruiz, J. Wang, H. Ksiezak-Reding, L. Ho, X. Qian, N. Humala, S. Thomas, P. Martínez-Martín, G. M. Pasinetti

**Affiliations:** ^1^Department of Psychiatry, Mount Sinai School of Medicine, New York, NY10029, USA; ^2^Alzheimer Disease Research Unit, CIEN Foundation-Reina Sofia Foundation, Valderrebollo 5, 28031 Madrid, Spain; ^3^GRECC, James J. Peters Veterans Affairs Medical Center, Bronx, NY 10468, USA

## Abstract

Epidemiological evidence suggests that hypertension may accelerate the onset and progression of Alzheimer's disease (AD). In this study, we explored the role of hypertension in the neurodegenerative changes associated with A*β* and tau aggregation. We induced hypertension in APP_swe_ Tg2576 and P301L-tauTg mouse models. In Tg2576 mice, experimental hypertension was associated with a significant increase of the accumulation of Amyloid-*β* (A*β*) peptides in brain tissue and a significant reduction of A*β* peptides in serum (*P* < .05). These results indicate that hypertension may promote AD-type A*β* neuropathology in Tg2576. In P301L-tauTg mice we found that the presence of hypertension was significantly associated with aggravated motor function assessed by hindlimb extension test (*P* = .01). These results suggest that hypertension may play a role in accelerating the progression of motor dysfunction associated with tau-related alterations. Our studies suggest that the management of blood pressure (BP) may alleviate AD-type A*β* neuropathology and neurological disorders associated with abnormal tau metabolism.

## 1. Introduction

As the population ages, the prevalence of AD dramatically increases and poses tremendous impact on the provision of health care and economy, apart from its devastating social implications. According to Ziegler-Graham et al. [1], if it was possible to delay the onset of AD by as little as one year, that would reduce the prevalence of AD by 12 million fewer cases worldwide in 2050. Therefore, it is urgent to identify effective strategies to prevent or delay the onset or to slow or reverse AD progression. Identification of risk factors is important for developing preventive strategies. Out of the many studies to identify potentially preventable risk factors, the most consistent finding has been the association of cognitive impairment with cardiovascular disease risk factors [2].

One of the most common cardiovascular risk factors is arterial hypertension, and several longitudinal studies suggest that BP levels are increased decades before the onset of AD and higher BP seems to accelerate the rate of cognitive decline in patients with early-onset AD [3, 4]. Furthermore, epidemiological evidence suggests that hypertension may promote the onset and progression of A*β* and tau neuropathology. Sparks et al. [5] associated hypertension with increasing incidence and severity of A*β* and tau neuropathology in the postmortem brain (e.g., parahippocampal gyrus) of nondemented individuals. 

Moreover, the Honolulu Heart Program/Honolulu-Asia aging study reported elevated BP in midlife associated with greater numbers of neuritic plaques in the neocortex and hippocampus and greater number of neurofibrillary tangles (NFTs) in the hippocampus [6], compared to the reference group.

Furthermore, the early epidemiological studies by Ghika and Bogousslavsky [7] showed that a transient phase of high BP occurred before the first motor symptoms in 80% of the patients clinically diagnosed with progressive supranuclear palsy (PSP). More recently, Papapetropoulus et al. [8] reported that the most common comorbid condition in PSP patients was hypertension, present in 50% of the PSP cases. However, Colosimo et al. [9] showed that prevalence of hypertension in pathologically proven PSP patients was no higher than in controls. Colosimo et al. [9] suggested that the apparent association between hypertension and PSP reported by Ghika might be due to the inclusion of multiinfarct/multilacunar states masquerading clinically as PSP. Because an association between brain vascular lesions, hypertension, and PSP is plausible, Sibon et al. [10] asserted that determining the exact frequency of hypertension in PSP patients will require careful, unbiased clinical diagnoses to distinguish “mixed” from “pure” PSP cases.

The controversy of the epidemiological data shows the need for clarification in order to properly address the implications of managing BP as prevention strategy of AD.

In an effort to clarify this controversy, we experimentally explored the potential causal relationship between high BP and the onset and/or progression of AD-type A*β* neuropathology and tau-mediated motor dysfunction. We examined the hypothesis that induction of hypertension in Tg2576 and P301L tau Tg mouse models would influence the onset and/or progression of AD-type A*β* neuropathology and tau-mediated phenotypes, respectively. 

## 2. Materials and Methods

### 2.1. Mouse Models

#### 2.1.1. Tg2576 Mouse Model of A*β* Neuropathology

We used female Tg2576 AD transgenic mice [11] obtained from Taconic Farms. Tg2576 mice express the human 695-amino acid isoform of the Amyloid precursor protein (APP) containing the Swedish double mutation (APP_Swe_) [(APP695) Lys670 → Asn, Met671 → Leu] driven by a hamster prior promoter, which leads to extracellular accumulation of both A*β* (1-40) and A*β* (1-42/43) peptides, as well as soluble high-molecular-weight A*β* oligomers and amyloid plaque deposition in cortical and limbic structures. 

#### 2.1.2. P301L-Tau Tg Mouse Model of Tau-Neuropathology

We used hemizygote female JNPL3 mice [12] obtained from Taconic Farms. We refer to these mice as P301L-tau Tg mouse model of tauopathy. The P301L-tau Tg mouse model expresses human tau with the most common FTDP-17 P301L mutation under the prion protein promoter. It is one of the best characterized mouse models of tauopathy and it is well accepted and considered to be an excellent in vivo model for studying consequences of tangle development. 

All animal studies were approved by the Institutional Animal Care and Use Committee of Mount Sinai School of Medicine.

### 2.2. Induction of Hypertension

Angiotensin II infusion method and Deoxycorticosterone acetate (DOCA)-salt method were used to induce essential hypertension in Tg2576 and P301L-tau Tg mice, respectively. These two methods model the two mechanisms underlying most of the cases of essential hypertension in humans.

#### 2.2.1. Induction of Chronic Hypertension Using Angiotensin II Infusion Method

We induced chronic hypertension in Tg2576 mice by infusing AngiotensinII. 

This experimental model system induces a fast and consistent elevation of the BP, appropriate for a short-duration treatment. In our studies, treatment lasted from 5 to 7 months of age, corresponding to the critical period during which an exponential accumulation of A*β* peptides occurs in Tg2576 mice.

Female Tg2576 mice were treated with Angiotensin II (Sigma 1.1 mg/Kg/day in 0.9% NaCl), infused for 8 weeks by means of 28-day micro-osmotic pumps (Alzet, Durect Corporation, Calif, USA, model 1004) implanted subcutaneously at the back of the neck. In control studies, age- strain- gender- matched Tg2576 mice were treated with 0.9% NaCl also infused by means of micro-osmotic pumps. The pumps were replaced once, after the first month of treatment.

Follow-up assessment included measurements of BP and body weight once every two weeks.

#### 2.2.2. Induction of Chronic Hypertension Using the DOCA-Salt Paradigm

We induced chronic hypertension in WT and P301L-tau Tg mice using the DOCA-salt method. 

This experimental model system progressively induces hypertension and was appropriate for a long-duration treatment, given the fact that symptoms of motor dysfunction slowly develop in the P301L-tau Tg mice. Mice were chronically treated with DOCA-salt for 8 months, starting at 5.5 months of age, 3 months before the first motor symptoms start in this mouse model, and ending at 13.5 months of age. 

Briefly, mice assigned to the hypertensive group underwent right unilateral nephrectomy at 20 weeks of age. After 2 weeks of recovery, 90-day-release DOCA pellets (50 mg/pellet, Innovative Research of America) were implanted subcutaneously by incision at the back of the neck. DOCA pellets were replaced every 3 months. Starting with the initial DOCA pellet implantation, mice in the hypertensive group received additional dietary salt chronically until the end of the study by incorporation of 1% NaCl into the drinking water.

Age-, strain-, and gender-matched P301L-tau Tg mice assigned to the control group also underwent the same procedure but were implanted subcutaneously with 90-day-release placebo pellets (50 mg/pellet Innovative Research of America). This treatment does not lead to detectable modifications in BP.

Follow-up assessment included measurements of BP and body weight once every two weeks.

### 2.3. Blood Pressure Measurements

BP measurements were conducted using a BP analysis system designed for small rodents according to manufacture's instruction (Hatteras Instruments, NC, USA). Specifically, mice were temporarily immobilized in a restraining chamber, the tail was inserted through the tail cuff and laid down into the tail slot, secured with a piece of tape. Every mouse underwent 5 preliminary cycles for which data was not recorded. The following 10 measurements of systolic, diastolic, and mean arterial pressure (MAP) were recorded and the mean values were used for each animal.

### 2.4. Behavioral Tests

#### 2.4.1. Assessment of Cognitive Function by Morris Water Maze Test

The Tg2576 mouse model is well known to develop progressive cognitive deterioration associated with accumulation of A*β* peptides in the brain with increasing age, starting at 6 months of age [11].

Therefore, spatial learning memory was assessed by the Morris water maze (MWM) behavioral test, as described previously [13] after two months of hypertension. In this assay, mice were tested in a 1.25 m circular pool filled with water mixed with nontoxic white paint (Dick Blick Art Materials). Mice were trained to mount a hidden/submerged (1.5 cm below water surface) escape platform (14 × 14 cm) in a restricted region of the pool. Spatial memory was assessed by recording the latency time for the animal to escape from the water onto a submerged escape platform as a function of the number of learning trials during the learning phase. The water maze activity was monitored with the San Diego Instrument Poly-Track video tracking system. 

#### 2.4.2. Motor Function Assessment of P301L-Tau Tg Mice

P301L-tau Tg mice are known to develop progressive motor impairment [14]. We assessed the motor function of these mice by the hindlimb extension assay. For that purpose, we established a scale from 1 to 4, which allowed us to clearly distinguish 4 different phenotypes (Figure 3(a)).

P301L-tau Tg mice were tested in the presence and absence of experimentally induced hypertension. To monitor hindlimb extension, mice were elevated by the tail by two different investigators who were blind to the treatment. The unimpaired animals, which generally place their legs in a wide “V” away from their bodies, were scored 4 and were identified as “normal phenotype.” We scored 3 when the mice were not able to completely separate the legs from the body, sometimes displaying a trembling leg, but with no dystonic symptoms. This group was considered to have “mild phenotype.” When dystonic symptoms were present, but the mice still had the ability to move their legs, we scored 2 and they were assigned the “moderate phenotype.” Finally, we scored 1 when the mice showed “severe phenotype”, with very dystonic hindlimbs, and were unable to perform any leg extension or movement when elevated by the tail. Each test score was the average of two trials of 3 and 5 seconds, respectively. We repeated the test twice the same day one hour apart, twice more the following day, and once one week later. The final score for each mouse was the average of the 5 tests. We monitored the hindlimb extension at 11 and 14 months of age (after 5 and 8 months of treatment, resp.).

### 2.5. Neuropathological Analysis

#### 2.5.1. Assessment of AD-Type Amyloid Neuropathology in Tg2576 Mice

For quantitative assessment of brain A*β* peptides, frozen pulverized tissue was homogenized in 5.0 M guanidine buffer, diluted (1 : 10) in phosphate-buffered saline containing 0.05% (v/v) Tween-20 and 1 mM Pefabloc protease inhibitors (Roche Biochemicals, Indianapolis, Ind, USA) and centrifuged for 20 minutes at 4°C. Total A*β*1-40 or A*β*1-42 in the supernatant was quantified by sandwich ELISA (BioSource, Camarillo, Calif, USA), as previously reported [15].

#### 2.5.2. Assessment of Tau Neuropathology in P301L-Tau Tg Mice

Abnormal accumulation of pathological tau species (64, 140, and 170 KDa) in the spinal cord of the P301L-tau Tg mice has been associated with the motor deficits developed in this mouse model [14, 16]. We followed Berger et al. procedures [16] to evaluate the presence of tau species in the spinal cords of both control and hypertensive P301L-tau Tg mice by Western blot. Briefly, spinal cords were homogenized in 10 × volume of the following mM: 50 mM Tris-HCl, pH 8.0, 274 mM NaCl, 5 mM KCl, 2 mM EGTA, 2 mM EDTA, protease inhibitor cocktail (Sigma, St. Louis, Mo, USA), phosphatase inhibitor cocktail I and II (Sigma). The homogenate was sonicated and spun for 15 minutes at 13 000 × g, and the supernatant was analyzed by Western blot (NFTs and cell debris were removed in the pellet). Proteins were separated on 10% SDS-PAGE Tris-glycine gels (Invitrogen) and wet transferred to nitrocellulose membranes. The membranes were blocked in 5% milk in PBST, and incubated with primary antibodies overnight. The following antibodies were used: PHF1 (anti-phospho-tau epitope S396-S404, 1 : 2000, generous gift from P. Davis), AT8 (anti-phospho-tau epitope S202-S205, 1 : 1000; Pierce, Rockford, Ill, USA), and anti-glyceraldehyde-3-phosphate dehydrogenase (GAPDH) (1 : 10 000, Chemicon International). Secondary antibodies (Santa Cruz) were used at 1 : 20 000 in 2% milk-PBST. Labelling was visualized using Western lightning Chemiluminescence reagent (PerkinElmer, Wellesley, Mass, USA). For densitometric analysis, ImageJ program was used. The intensity of the protein band of interest was divided by the intensity of the band representing a loading control GAPDH to calculate the relative amount.

### 2.6. Statistical Analysis

All values were expressed as mean ± standard error of the mean (SEM). Differences between means were analyzed using two-tailed Student's *t* test *versus *normotensive-control mice. In all analyses, the null hypothesis was rejected at the 0.05 level. All statistical analyses were performed using the Prism Stat program (GraphPad Prism 4 Software, Inc., San Diego Calif, USA).

## 3. Results and Discussion

### 3.1. Experimental Hypertension Models Significantly Elevated Blood Pressure in Tg 2576 and P301L-Tau Tg Mice

**Figure 1:**
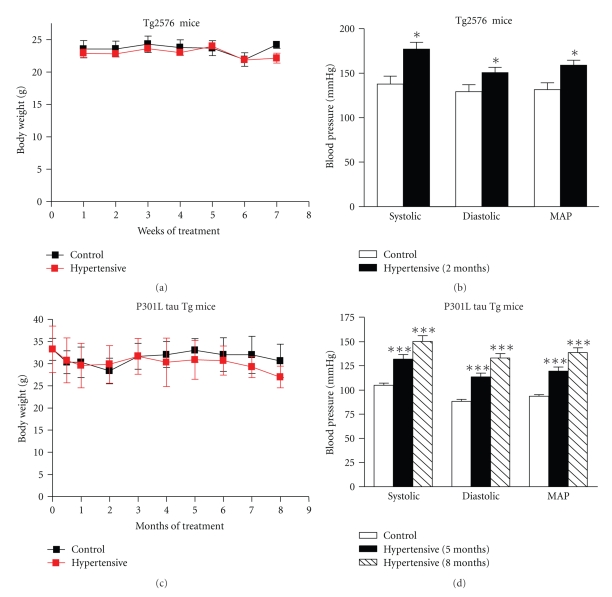
*Experimentally induced hypertension significantly elevated the blood pressure of Tg 2576 and P301L-tau Tg mice. *(a), (c) Experimental hypertension had no aversive effect on body weight of mice *versus* normotensive control group. (b), (d) Experimental hypertension progressively elevated BP as shown by systolic, diastolic and mean arterial pressure (MAP). Values represent mean ± SEM values. (a), (b) *n* = 6 mice per group. (c), (d) *n* = 10 mice per group. Differences between means were analyzed using two-tailed Student's *t* test *versus* control normotensive mice, *P < .05, ***P < .0001 by two-tailed Student's *t* test analysis.

Experimental hypertension had no aversive effect on body weight of mice *versus* normotensive control mice (Figures 1(a) and 1(c)).

Angiotensin II treatment significantly increased BP of Tg2576 mice by 20% (*P* = .02), as shown by the systolic (28.4% increase), diastolic (16.6% increase) and MAP (20.8% increase) values (Figure 1(b)). Elevation of MAP was reached after two weeks of treatment and was consistently maintained over the two-month duration of the experiment. DOCA-salt method of hypertension induction was not used in this specific study because DOCA-salt method increases the liquid uptake and urine secretion which will disrupt the homeostasis balance of amyloid peptides between blood and brain.

Also, DOCA-salt method progressively induced chronic hypertension in the P301L-tau Tg mice compared to the placebo-salt group (control group) as indicated by systolic, diastolic and MAP values (Figure 1(d)).

After one month of treatment, the MAP of the DOCA-salt treated mice (hypertensive group) was already significantly elevated, by 10% (*P* = .01 data not shown). From 3 to 5 months of DOCA-salt treatment, MAP values reached a 30% increase* versus* placebo-salt treatment (*P* < .005, Figure 1(d)). By the end of the study, after 8 months of DOCA-salt induced hypertension, the MAP reached 40% higher values than controls (*P* < .0001, Figure 1(d)).

The DOCA-salt method induced elevation of BP in WT mice in a similar pattern as in P301L-tau Tg mice.

### 3.2. Hypertension May Have a Causative Effect in Promoting A*β* Neuropathology of AD-Type

**Figure 2:**
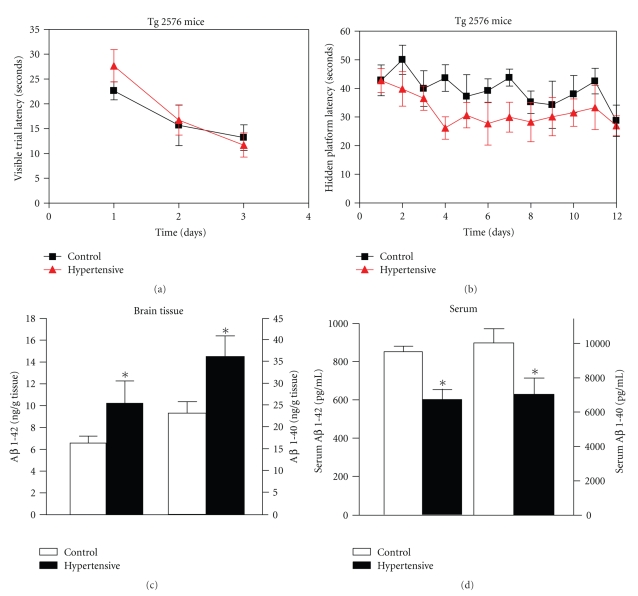
*Hypertension promoted AD-type* *A* *β* *neuropathology in Tg2576 brains. *(a), (b) Assessment of spatial memory in hypertensive Tg 2576 mice *versus* control normotensive mice by MWM test showed no effect of hypertension in the inability to learn of Tg 2576. Latency score represents time taken to escape to the platform from the water. (a) Visible platform acquisition. (b) Hidden platform acquisition. (c) A 2-month period of hypertension significantly increased the accumulation of A*β*1-40 and A*β*1-42 peptides by 57 and 51%, respectively, in brain tissue of Tg2576 mice. (d) This increase of Ab peptides in the brain was accompanied by a significant 30% reduction of both A*β*1-42 and A*β*1-40 peptides in the serum of Tg2576 mice. Values represent mean ± SEM values, *n* = 6 mice per group. Differences between means were analyzed using two-tailed Student's test *versus* control mice, *P < .05.

ELISA analysis showed that experimental hypertension significantly increased by 57%, the accumulation of A*β*1-42 peptide (*P* = .01) in brain tissue relative to age-, strain-, and gender-matched control Tg2576 mice (Figure 2(c)). Accumulation of A*β*1-40 in brain tissue was also increased, by 51% (*P* = .02), compared to control animals (Figure 2(c)). Increase of A*β* peptides in the brain was accompanied by a significant 30% reduction of both A*β*1-42 and A*β*1-40 peptides in the serum of these animals (*P* = .02, Figure 2(d)). 

Our finding of aggravated A*β* load in brain tissue of hypertensive Tg2576 mice experimentally supports previous epidemiological Honolulu Heart Program/Honolulu-Asia aging study [6], which had demonstrated an association between midlife elevation of BP and A*β* deposition in the neocortex and hippocampus of AD patients.

We hypothesized that accumulation of A*β* peptides in brain of hypertensive mice, concomitant to the decrease of A*β* peptides in serum of these animals could be due to the dysregulation of A*β* trafficking in and out of the brain. Our hypothesis is supported by the data reported by Gentile et al. [17], showing that induction of hypertension for one month in WT mice resulted in a significantly enhanced deposition of A*β* in the hippocampus and cortex. Furthermore, the authors associated the enhanced amyloid deposition with abnormally increased blood-brain barrier (BBB) permeability of blood-born substances in the cortex and hippocampus, suggesting a bloodstream origin of the brain amyloid deposits, rather than a neuronal source. These studies correlate well with our finding of increased levels of A*β* peptides in the brain and decreased levels in serum. However, in addition to changes in BBB permeability, we cannot rule out the possibility of dysregulation of A*β* transporters affecting the trafficking of A*β* peptides from the brain to the serum, decreasing the clearance of A*β* peptides from the brain of Tg2576 mice. 

Consistent with previous results [11], control Tg2576 mice at 7 months of age showed impaired acquisition of spatial learning, as assessed by MWM test. The mice failed to learn to use the available visual cues to help locate a submerged escape platform, as indicated by the lack of significant improvements in the escape latency across consecutive learning sessions (Figures 2(a) and 2(b)). Similarly, hypertensive strain-, age-, and gender-matched Tg2576 mice were also unable to locate the escape platform (Figures 2(a)  and 2(b)). We did not observe a significant difference in the spatial learning ability by the MWM test between the two groups. 

Hypertension induced by angiotensin II infusion for 8 weeks in Tg2576 mice significantly increased the content of total A*β* peptides in the brain compared to normotensive Tg2576 control mice. However, we failed to identify any significant alteration in spatial memory function. It is possible that hypertension induced by angiotensin II increased total A*β* content without significant impact on the amount of oligomeric A*β*, which is largely responsible for cognitive deterioration in the mouse model of AD. It is also possible that the degree of hypertension imposed to the mice is quite mild (elevation of BP by 20%). More severe hypertension condition might be needed to cause worse cognitive function in this mouse model.

### 3.3. Chronic Hypertension May Have a Role in Aggravating the Progression of Tau-Mediated Motor Impairment

As mentioned before, the P301L tau Tg mice, so it reads P301L Tg mice progressively develops motor impairment over an extended period of time. Hypertension induction by Angiotensin II infusion through micro-osmotic pump requires surgical implants every 28 days. Each surgical maneuver puts animals under stress and sometimes mortality. Therefore, we used the DOCA-salt method which only needed replacement of the implants three times over the 8 months of treatment.

**Figure 3:**
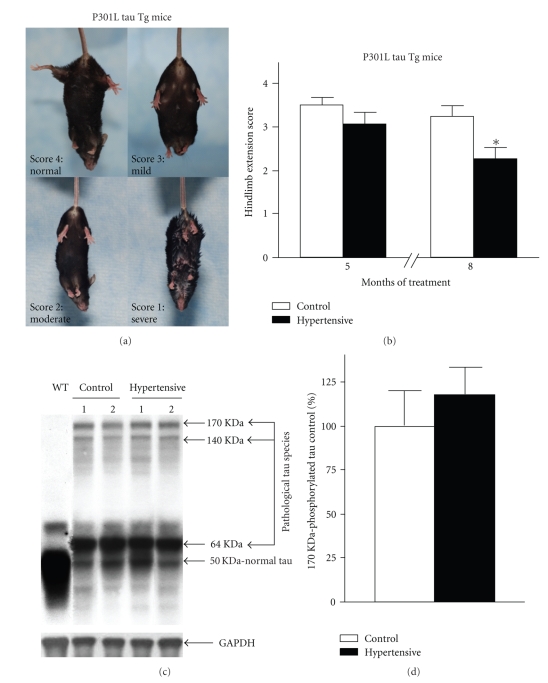
*Chronic hypertension aggravated motor deficits assessed by hindlimb extension test in P301L-tau Tg mice. *(a) Score scale used to assess motor function of the P301L-tau Tg mice by the hindlimb extension test. (b) Motor function assessed by hindlimb extension test of P301L-tau Tg mice was not affected after inducing hypertension for 5 months (11 months of age), but was significantly aggravated, by 30%, after inducing hypertension for 8 months (14 months of age). Score values represent mean ± SEM of 5 tests. Also *P < .01, by two-tailed Student's *t* test analysis. (c), (d) Hypertension did not significantly alter tau neuropathology in spinal cords of P301L-tau Tg. (c) Representative Western blot for tau species of WT and P301L-tau Tg (control and hypertensive) spinal cord samples. GAPDH was used as a loading control. (d) Densitometric quantification of the 170 KDa tau species levels in spinal cord samples. Values represent mean ± SEM values. *n* = 10 mice per group. Differences between means were analyzed using two-tailed Student's *t* test *versus* control mice.

The control P301L tau Tg mice assessed at 11 months of age by hindlimb extension test showed “normal phenotype,” according to the score scale that we defined to distinguish the different phases of the motor impairment mediated by tau (Figure 3(a)). Thus, the average hindlimb extension score of the control group was 3.49 ± 0.17 (Figure 3(b)). 

At the same age, after 5 months of hypertension induction P301L tau Tg mice displayed “mild phenotype,” as indicated by an average score for the hindlimb extension test of 3.06 ± 0.25 (Figure 3(b)).

Statistical analysis of the data using the two-tailed Student's *t* test showed no significant differences between the control and the hypertensive P301L-tau Tg groups on motor function assessed by the hindlimb extension test at 11 months of age. These results indicate that 5 months of experimental hypertension did not significantly affect the motor function of P301L-tau Tg mice (Figure 3(b)). 

At 14 months of age the control P301L-tau Tg mice had “mild phenotype,” as shown by an average score in the hindlimb extension test of 3.24 ± 0.24. 

Interestingly, at this age, after 8 months of hypertension, the average hindlimb extension score of the hypertensive group had dropped down to 2.22 ± 0.29, identified as “moderate phenotype.” Most importantly, the hypertensive P301L-tau Tg group showed significantly worsened phenotype, by 30%, compared to age-, strain-, and gender-matched normotensive control animals at this stage of the study (Figure 3(b), *P* = .01).

Indeed, compared to control P301L-tau Tg mice, hypertensive P301L-tau Tg mice showed significantly greater leg weakness, dystonic posturing and hindlimbs with clear tendency to clasp. Consistent with the observation that hypertension is associated with more severe motor impairment in P301L-tau Tg mice, motor impaired hypertensive mice were also characterized by poor grooming (urine scalding/oily fur, not shown). We only occasionally observed dystonic hindlimbs in the age-, strain-, and gender-matched control mice. None of the control P301L-tau Tg mice had problems with grooming. 

At the end of the study, at 14 months of age, motor function scores of P301L-tau Tg mice showed a significant negative correlation between systolic BP (*P* = .044, *r* = 0.45, *R* ^2^ = 0.2), and MAP values (*P* = .046, *r* = 0.45, *R* ^2^ = 0.2), suggesting that hypertension may be promoting clinical motor impairment.

The motor disturbances developed by P301L-tau Tg mice have been correlated with the presence of abnormally hyperphosphorylated tau species in the spinal cord of these mice [14, 16]. Therefore, we proceeded to evaluate whether hypertension influenced tau neuropathology present in spinal cords.

Western blot analysis of spinal cords from WT mice showed normal 50 KDa tau species (Figure 3(c)). Spinal cords of control P301L-tau Tg mice showed abnormal accumulation of 64, 140, and 170 KDa pathological tau species (Figure 3(c); 14, 16). Quantitative analyses of pathological tau species found in control P301L-tau Tg samples showed a negative correlation with hindlimb extension scores (*P* = .01, *r* = 0.7, *R* ^2^ = 0.5). Our data support the hypothesis that motor impairment developed by P301L-tau Tg mice may be mediated by the abnormal tau species.

However, densitometric analysis of Western blot tau species (50, 64, 140, and 170 KDa) present in spinal cords of hypertensive *versus* control P301L-tau Tg mice did not detect a significant increase in the immunoreactivity of any of the tau species (Figure 3(d)), as shown for the representative 170 KDa tau species. These results indicate that the aggravated motor disturbances associated with induced chronic hypertension were not mediated by tau neuropathology.

Alternatively, we think that responsible factors could be vascular and/or white matter lesions [3, 18, 19], occurring with long history of hypertension. Future studies in our lab will be addressing the effect of hypertension in these lesions and whether or not they influence the progression of the motor deficits in a tau-independent but convergent manner.

On the other hand, it is also very likely that the motor function of the P301L-tau Tg mice assessed by hindlimb extension only becomes significantly sensitive to hypertension when the clinical progression of the disease reaches a certain threshold. In agreement with the possible existence of a “critical point” in the development of behavioral deficits by P301L-tau Tg mice, Morgan et al. [20] have recently reported that hindlimb motor paralysis secondary to tau aggregate formation did not exhibit antecedent deficits in motor behavior prior to the onset of paralysis.

While the mechanistic basis for the aggravated motor phenotype associated with chronic hypertension in P301L-tau Tg mice is not clear, our observation suggests that it may be necessary to reexamine epidemiological data showing that hypertension was present in a high percentage of PSP patients [7, 8] to separately explore “mixed cases” with vascular lesions from “pure PSP cases,” for a better understanding of the actual association between hypertension and movement disorders mediated by abnormal metabolism of tau. 

Furthermore, the interaction between A*β* and tau neuropathology may be necessary to modify the “critical point” in an additive manner or in an order of magnitude greater than in a simple additive effect. Therefore, further studies in double transgenic mouse models showing both A*β* and tau neuropathologies are required to fully address the question on the role of hypertension in dementia of AD-type.

Taken together, our studies suggest that BP management may have a beneficial effect delaying and/or attenuating A*β* neuropathology present in AD patients, and may also attenuate the increasingly severe progression of the movement disorders present in patients with tauopathies.
